# Ruminative and mood associations for age differences in social and directive reasons to think and talk about life experiences

**DOI:** 10.1371/journal.pone.0235378

**Published:** 2020-07-16

**Authors:** Jorge J. Ricarte, Laura Ros, Jose M. Latorre, Tom J. Barry

**Affiliations:** 1 Department of Psychology, University of Castilla-La Mancha, School of Education, Albacete, Spain; 2 Department of Psychology, University of Castilla-La Mancha, School of Medicine, Albacete, Spain; 3 Department of Psychology, The University of Hong Kong, Hong Kong, Hong Kong; 4 Department of Psychology, The Institute of Psychiatry, Psychology & Neuroscience, King’s College London, London, United Kingdom; National University of Ireland Maynooth, IRELAND

## Abstract

Reminiscing, or thinking and talking about our past experiences, can have mood enhancing effects. Rumination is implicated in reminiscence and yet has been shown to have negative effects on mood, with important differences between age groups. However, age differences in the effects of reminiscing on mood, and particularly the effects of rumination within reminiscence, are less explored. Two different age groups completed self-report measures of the positive directive (planning for present and future behaviors) and social (communication) uses of autobiographical memory, as well as maladaptive rumination and depression symptom severity. Young participants (Mean age: 21.82) ruminated more and reported greater frequency of using the directive and social functions of thinking and talking about past experiences than older adults (Mean age: 71.82). These reminiscence processes were also differentially associated with depression symptoms between age groups when tested in structural equation models. In older participants, but not young participants, the directive function was negatively associated with depression severity; in young participants, but not old participants, the social function was negatively associated with depression severity. Furthermore, although depressive and abstract rumination was directly positively related to depression scores in both samples, this association was inverted when the effect of rumination on depression was calculated through functions of reminiscence. The implications of these results for intervention development in older versus young adults, is discussed.

## Introduction

Thinking and talking about our past autobiographical experiences, or reminiscing, has been proposed to serve a number of functions that each influence emotional wellbeing [1] [2] [3]. Although many of these functions are thought to enhance wellbeing, some of them are suggested to be detrimental [4]. Importantly, there is a substantial degree of conceptual overlap between some of these negative reminiscence functions and another depression-linked concept, rumination, or repetitive thinking about the causes and consequences of one’s negative mood [5]. Furthermore, the functions of reminiscence and the use of rumination have both been found to differ across the lifespan [6] [7] [8]. In addition, reminiscence therapy has positive effects on depression, self-esteem, integrity, life satisfaction, and psychological well-being [9]. However, to our knowledge, no study has yet examined age-related differences between young and older adults, in the interaction between reminiscence functions and rumination and their relations with wellbeing-related outcomes such as depression symptom severity.

Webster [10] suggested eight functions for reminiscence that are each hypothesised to be associated with wellbeing: bitterness revival, boredom reduction, conversation, death preparation, identity, intimacy maintenance, problem solving and to teach/inform. Subsequently, in an attempt to create a more integrative theory of the functions of autobiographical memory, Bluck & Alea [11] suggested three overarching functions: self, social, and directive. Memories can be used to help people maintain a sense of being the same person over time (Self; [12]), to develop, maintain, and enhance social bonds (Social; [13]) and to direct present problem solving and future behaviours (Directive; [14]). Research indicates that utilisation of the social and directive functions is typically associated with positive emotional outcomes [15]. However, utilisation of the self-function has been found to be associated with heightened depression severity [16]. In particular, Grace et al. [16] found that people who thought a lot about their past, but did not discuss it with others, showed particularly high depression severity. In addition, people who tried to recall past events in order to maintain a sense of self-continuity across time, perhaps to compensate for a weakened sense of self-continuity, also showed higher depression severity. Other research utilising Webster’s [10] original framework for reminiscence functions has also found that some self-relevant functions, such as bitterness revival or the tendency to re-experience past resentments, have also been associated with elevated depression symptomatology [4].

It is possible that people who often use their memories to serve the self-function may be driven to do so in response to their tendency to ruminate on the causes and consequences of negative moods. People who engage in this kind of ruminative thinking often dwell on abstract and unconstructive questions such as “why do bad things always happen to me?”. A person may subsequently retrieve autobiographical memories to either reinforce or challenge their negative self-view. Indeed, research indicates that there is an association between the tendency to engage in rumination and the utilisation of the negatively valenced functions of reminiscing [17]. Rumination use has also been found to predict greater use of bitterness retrieval [18]. As suggested by Cappeliez et al., [6] people who tend to engage in self-focused rumination in response to negative moods may retrieve and re-experience unresolved disturbing events from their past that align with this negative interpretation of themselves, and together this thinking style may serve to maintain their depression symptoms. In addition, otherwise positive reminiscence functions, such as intimacy maintenance, can lead to negative emotional states if one also engages in self-focused rumination [19].

Rumination about the past can have positive and negative effects on emotions and psychopathological symptoms depending on the nature of one’s rumination (e.g., the content and duration of one’s ruminative thoughts). Watkins [5] suggests that there are substantial differences, in terms of emotional consequences, between unconstructive ruminations that are abstract and concern one’s emotional responses to experiences (e.g. Why did I feel this way?) and constructive ruminations that focus on the concrete details by which events occurred (e.g. How did that happen?). Unconstructive rumination use is associated with social problem solving impairments in depressed patients [20] and anomalous perception of reality (e.g. perceived stimuli in the absence of a real source) in clinical [21] and non-clinical samples [22]. Although rumination can be initially used as an adaptive cognitive strategy to control distress and gain insight about emotions generated by a distressing situation, the continued use of rumination may lead to the perpetuation of mood disturbances [23].

Although ruminative tendencies have been found to be associated with reminiscence functions, both processes have been found to differ across the lifespan. In particular,older adults report a lower use of rumination than younger participants [8]. Compared to younger adults, older adults have also been found to self-report that they rely less on the social function of reminiscence, as measured by the Thinking about Life Experiences (TALE) questionnaire [24]. This is in accordance with suggestions regarding socio-motivational changes that occur across the lifespan [25]. In young adults, initiating relationships and creating interpersonal bonds are of utmost importance whereas older adults are less motivated to initiate new relationships and instead focus on those that have already been acquired [26]. In addition, young adults report that they rely more on the self and directive functions of reminiscence, compared to older adults, perhaps because of their relative lack of experience regarding the management and understanding of self-relevant thoughts and feelings [27] and the critical decisions they must make about their future professional and personal life goals [28].

Given the theoretical and empirical association between rumination and reminiscence and the importance of these processes in predicting depression symptomatology, it is of utmost importance that we understand how changes in these processes over the lifespan are also associated with changes in their associations with one another and with depression. In accordance with previous research, we expect that older adults will ruminate less and report lower use of the social and directive functions of reminiscence than young adults. Secondly, young adults are expected to rely more on social functions of reminiscence to regulate emotions than older participants. Finally, the interaction between rumination and reminiscence functions are expected to show positive effects on emotional status in both age groups.

## Materials and methods

This study was carried out and approved in accordance with the recommendations of Acta number 06/2016 of the Clinical Research Ethical Committee (CEIC) from regional Health Service of Castilla la Mancha, Spain. All subjects gave written informed consent in accordance with the Declaration of Helsinki.

### Participants

Young and older adults were recruited using available resources of the senior author’s institution (web pages, flyering, press notes). In addition, a snowball sampling method was also used whereby participants were asked to encourage relatives and acquaintances to enrol. Participants who reported currently receiving treatment for mental illness were excluded. In addition, the group of older participants completed the Mini Mental Status Examination [29] so that participants with cognitive impairment could be excluded. Two hundred fifteen young (Mean age: 21.82, SD: 6.23) and 219 older (Mean age: 71.82, SD: 10.34) adults participated in the study. These groups were balanced in terms of gender (52.1% young women, 51.4% older women) and educational level (97.6% young and 84.5% older participants completed secondary studies) distribution.

### Measures

#### Directive and social functions of autobiographical memory

The use of autobiographical memory for directive and social purposes, was measured using the original Thinking about Life Experiences scale (TALE [30]). Instructions for the questionnaire read: “Sometimes people think back over their life or talk to other people about their life—it may be about things that happened quite a long time ago or more recently. We are not so interested in the times that you think back over specific events as in when and how you bring together and connect the events and periods of your life”. The items of the TALE concerned the directive (e.g., “I think or talk about past experiences when I want to learn from my past mistakes”) and social (e.g., “when I want to develop a closer relationship with someone”) functions of autobiographical memory. Responses regarding the frequency of use of each memory function were made on a 6–point Likert–type scale, ranging from *never* (1) to *very frequently* (6). Both the directive and social factors showed good internal consistency in the current data (Cronbach alpha: .82 and .87, respectively).

#### Rumination

Depressive rumination was measured with the 4-item short version of the Leuven Adaptation of the Rumination on Sadness Scale (LARSS; [31]). This scale concerns the lack of controllability respondents have over negative thoughts. Participants were requested to rate on a scale of 0 (*never*) to 10 (*very often*) how often they experienced the following situations when feeling sad, down or depressed: (a) “I have difficulty getting myself to stop thinking about how sad I am; (b) “I get absorbed in thinking about why I am sad and find it difficult to think about other things”; (c) “I repeatedly try to figure out, by doing a lot of thinking, what might be the causes of my sadness”; and (d) “I keep thinking about how I feel, to understand myself and my sad feelings better”. Abstract rumination focused on verbal concepts and self-attributions was measured by means of the unconstructive factor from the Mini Cambridge-Exeter Repetitive Thought Scale (Mini-CERTS) [32]. Participants reported the frequency with which they ruminate using self-negative content (e.g. I compare myself to other people or I think I´m no good at all). Both scales showed good internal consistency for the current data, Cronbach alpha: .93 and .71, respectively.

#### Depression symptoms

Self-reported depression symptom severity was assessed with The Beck Depression Inventory Version II (BDI-II; [33]), a 21-item questionnaire where participants report their experience of typical cognitive, affective and somatic depressive symptoms within the past two weeks. Higher scores reflect worse depressive symptoms. The BDI-II showed strong internal consistency (α = .88).

### Procedure

After obtaining their sociodemographic information and age criteria fulfilment, participants were informed regarding the general information, duration, aims and types of tests to be completed within the study. Information about confidentiality and withdrawal were also given to participants. After obtaining informed consent, all measures were administered to participants in small groups (maximum of 20 participants per group) in different classrooms of the first author’s institution, supervised by one of the authors or a trained research assistant. The questionnaires were then administered in a single 30–45 min session starting with the TALE followed by BDI and rumination scales in a fixed order. Participants were not compensated for their time.

### Statistical procedure

In addition to T-tests for mean group differences in dependent variables and product-moment Pearson correlations between variables by group, path analyses were evaluated using the AMOS 19.0 software package. A maximum likelihood estimator was used to estimate all model parameters. In order to evaluate the fit of the models, we used χ^2^ statistic, the Comparative Fit Index (CFI; [34] [35]), and the root-mean-square error of approximation (RMSEA; [36]). According to Bentler [34], CFI values greater or equal to .90 are indicative of an acceptable fit. With regards to RMSEA, values lower or equal to .08 represent a reasonable fit [37]. With regards to χ2, a non-significant χ2 has been considered indicative of good fit [37].

The indirect effects of rumination (depressive and abstract scores were used to create the latent variable “rumination”) on depression symptoms via directive and social functions of autobiographical memory were examined with the bootstrapping sampling procedure [38]. Bias-corrected 95% confidence intervals on 5000 bootstrap samples were estimated for all direct and indirect effects. If the confidence interval did not include zero, the effect was significant.

In the young group, the model power value calculated for the RMSEA statistic [39] with the current sample size (n = 215) and with the number of variables introduced in the model (df = 2) was 0.99. In the older group the model power (n = 219 and df = 2) was also 0.99.

## Results

### Age differences in rumination and reminiscence forms

As can be shown in Table 1, T-test analyses showed that although older participants reported significantly higher BDI scores than their young counterparts, young participants reported a higher use of autobiographical memory functions and rumination forms than did older participants.

**Table 1:** Differences in dependent measures by age group (pending change to T-test).

	Young	Older		T-Test	Cohens D
	*M* (*SD*)	*M* (*SD*)	*F*	*p*	
**Directive**	44.84 (6.09)	36.79 (7.87)	137.84	< .001	1.14
**Social**	33.13 (6.99)	28.37 (8.20)	41.65	< .001	.09
**Depressive rumination**	14.78 (10.77)	11.97 (10.82)	7.27	.007	.02
**Abstract rumination**	21.11 (4.21)	18.97 (4.62)	24.35	< .001	.05
**Depression symptoms**	9.67 (7.25)	12.85 (9.03)	15.70	< .001	.04

Product-moment Pearson correlations showed no significant correlations between the use of autobiographical memory functions (directive or social) and depression symptoms scores (see Table 2). However, depressive and abstract rumination were positively and significantly correlated to both autobiographical memory functions for both young and older adult participants.

**Table 2:** Pearson correlation between measures (young participants above, older participants below).

	1	2	3	4	5
1. Directive	-	.54**	.13	.23**	.36**
2. Social	.48**	-	.09	.28**	.30**
3. Depression	-.10	.02	-	.49**	.38**
4. Depressive rumination	.19**	.27**	.52**	-	.36**
5. Abstract rumination	.23**	.27**	.39**	.59**	-

** = p < .01

### Path analyses results

**Fig 1:**
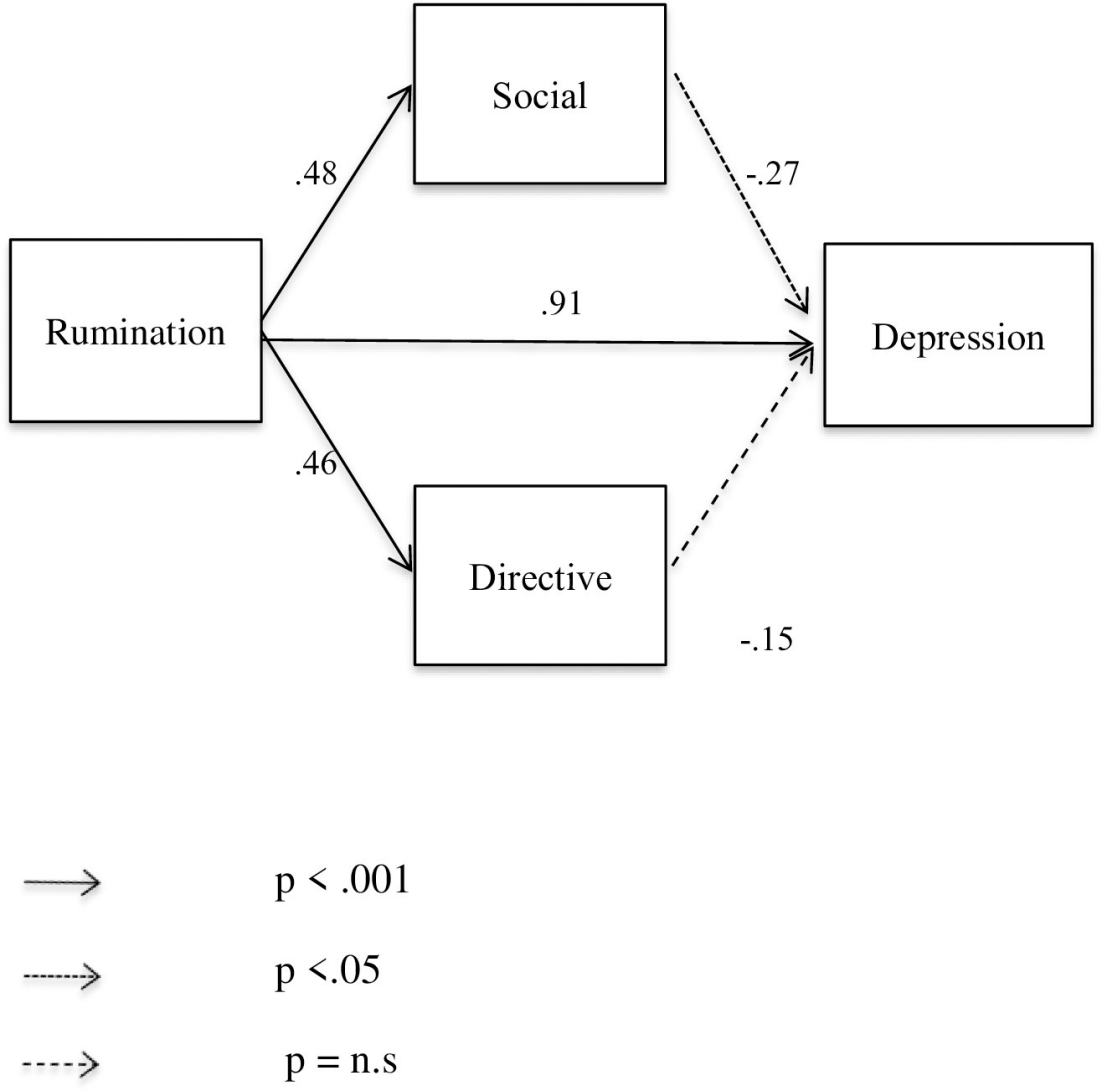
Path model with standardized regression weights of rumination on depression through social and directive functions in young participants.

**Fig 2:**
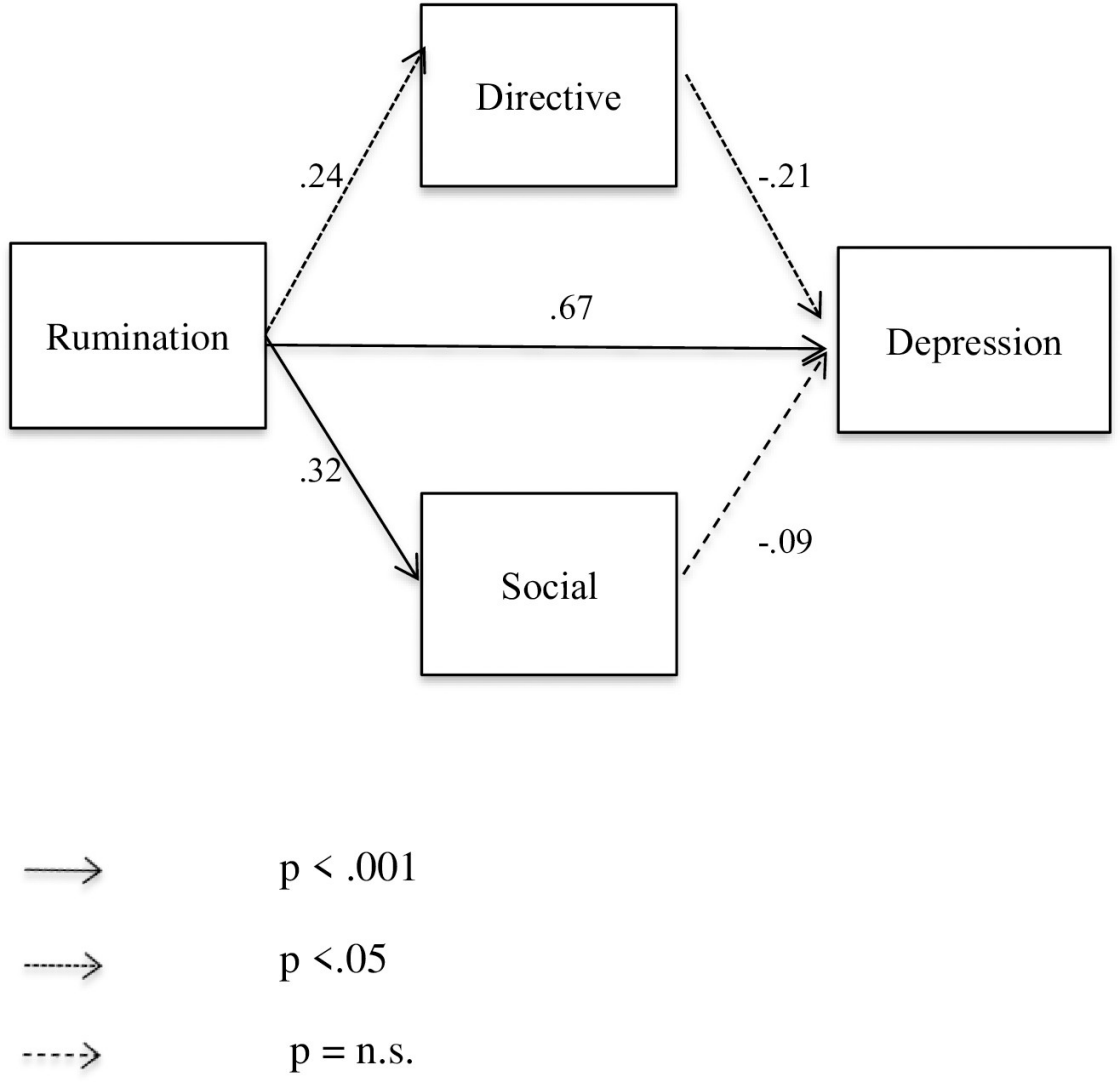
Path model with standardized regression weights of rumination on depression through social and directive functions in older participants.

The tested models in young (see Fig 1) and older (see Fig 2) adults showed good fit ((*χ*^*2*^(2) = 2.58, *p* = .075; CFI = .98, RMSEA = .08, and *χ*^*2*^(2) = 1.06, *p* = .346; CFI = .99, RMSEA = .01, respectively). Each direct effect between variables was significant except for the association between depression scores and the directive function in young participants and between depression scores and the social function in older participants. Most interestingly, the results of this model (rumination, autobiographical functions and depression scores) showed that when rumination is included in the equation, the previously non-significant associations between autobiographical functions and depression scores obtained with Pearson correlations (Table 2) changes. In particular, the directive function is then negatively associated with depression for both young and older participants.

In addition, the indirect effect in the model showed that rumination had a significant indirect negative effect on depression via the functions (directive and social) of autobiographical memory regardless of age (standardized indirect effect in young participants = -.195 [BC 95% CI = -.504, -.071], *p* = .001, and standardized indirect effect in older participants = —.079 [BC 95% CI = -.167, -.024], *p* = .004, respectively).

## Discussion

Confirming previous work on age differences in the functions of autobiographical memory, our sample of young participants, compared to older participants, engaged in a greater use of what are typically considered to be negative cognitive emotion regulation strategies (rumination). However, greater use of reminiscence functions and rumination in young participants, could be interpreted in terms of their life stage and not necessarily in terms of psychopathology (in fact older adults presented higher scores on depression symptoms). As previously argued, young adults have more decisions to make about their future and they are less experienced in how to use ones life experiences to enhance their socioemotional wellbeing [40]. From a developmental perspective, young adults need to spend time making sense of and learning from their experiences in order to develop emotionally and consolidate newly acquired relationships whereas for older adults, this emotional development is likely to have already taken place and their relationships are likely to already have been acquired and consolidated. The current results also seem to contradict theoretical approaches regarding the frequency with which older people engage in reminiscence, compared to young adults (e.g. [3]). Here, young participants reported a greater use of the directive and social functions of reminiscence than older participants and showed lower depression scores, although young participants also showed higher levels of depressive rumination compared to older participants. Emotional adjustment was operationalized as lower depression scores despite higher levels of depressive rumination compared to older participants. However, this last finding should be interpreted with caution as the reasons to older adults may have higher scores on the BDI-II compared to young adults because of the presence of items in this scale related to sleep, sex drive and appetite, which are likely to be reduced amongst older participants irrespective of their depressive symptomatology. Thus, a higher BDI-II score in older adults than in young participants in the current research is not necessarily an indicator of clinical depression.

Although previous research has attempted to associate autobiographical memory functions with psychopathology (especially depression), our results also suggest that one could separate autobiographical memory functions from the emotion generated by a concrete autobiographical memory. The use of autobiographical memories for mood-enhancement is more dependent on their emotional (positive) valence rather than their social or directive function [41]. Moreover, interventions that improve mood in major depression can enhance autobiographical memory [42]. However, this approach to autobiographical memory changes when rumination is considered. Compared to previous research where there was only a bivariate analysis of autobiographical functions, our current results showed an association between autobiographical memory functions that differed as a function of age and a negative association between rumination and depression scores through these autobiographical memory functions. Thus, one might conclude that autobiographical memory functions, in concert with rumination, can be used to reduce depression symptoms and that this effect might be most prominent for social functions in young people and directive functions in older people.

The rumination measures included in the present study were designed to capture maladaptive cognitions (depressive and unconstructive/abstract) and as such they showed a direct association with depression scores. However, the indirect effects in the structural equation model showed that that kind of rumination, when used to think and talk about past events, can have a positive mood-enhancement effect. Although previous research has shown that concrete rumination can be used to reduce depression [43], the current results suggest that abstract rumination may be used to gain insight about experiences needed for planning for present and future behaviors (directive function) and communication/sharing with others (social function). However, these results must be taken with caution as participants in the current study were not drawn from a clinical population and so may differ in the contents and duration of rumination as compared to clinical groups. To our knowledge, this is the first work showing that rumination might interact with the functions of autobiographical memories. Regardless of one’s age, rumination can be positively used to decrease depression symptoms when used to think or talk about social and directive functions of reminiscence. Future research should explore the link between ruminations about autobiographical memories with meaning making mechanisms included in narratives throughout the lifespan (e.g. [44]).

The current research has several important limitations. First, this investigation is correlational and so conclusions about causality are hypothetical. The current results should be tested with an experimental study where the impact of induced rumination about social and directive autobiographical experiences on depression is measured. Secondly, although we used a developmental approach to explain age differences, we did not include a middle aged group of people, limiting our conclusions about the trajectory of how these processes and their associations with one another change as people age and develop. Finally, although it was beyond the aims of the current research, the lack of clinically diagnosed depressed participants precludes us from generalising our results to people who experience more severe depression symptoms.

In conclusion, in addition to age differences in the frequency of use of different forms of autobiographical memory to adjust one’s emotional state, it is also possible that both younger and older people can use depressive and abstract rumination via those functions to decrease their depression symptoms.
