# Supplementary material for: Ruminative and mood associations for age differences in social and directive reasons to think and talk about life experiences
Source: PLoS One. 2020 Jul 16;15(7):e0235378. doi: 10.1371/journal.pone.0235378 (PMC7365414; doi:10.1371/journal.pone.0235378)
Supplement: Attachment — Submitted filename: Response to Reviewers 1.docx [file pone.0235378.s001.docx]

Dear Editor,

After submitting our first revised version of our article “PONE-D-19-31461 Ruminative and mood associations for age differences in social and directive reasons to think and talk about life experiences”, the article was directly rejected. However, we considered that we could fully address reviewers concerns as they were minor changes. Following instructions by Kate Massingham we present here a rebuttal letter including our point-by-point response to the original reviewers’ concerns (round 1) as well as a point-by-point response to the reviewer and Editor improvements needed in their second review (round 2)

PONE-D-19-31461
Ruminative and mood associations for age differences in social and directive reasons to think and talk about life experiences

**RESPONSES TO REVIEWERS: FIRST ROUND**

PONE-D-19-31461
Ruminative and mood associations for age differences in social and directive reasons to think and talk about life experiences

Dear Andrew Allen

Thank you for your positive feedback. We have ellaborated the demanded documents. Te “revised manuscript with track changes” includes performed changes highligthed in yellow

Journal Requirements:

1. When submitting your revision, we need you to address these additional requirements.
Please ensure that your manuscript meets PLOS ONE's style requirements, including those for file naming. The PLOS ONE style templates can be found at
http://www.journals.plos.org/plosone/s/file?id=wjVg/PLOSOne_formatting_sample_main_body.pdf and <http://www.journals.plos.org/plosone/s/file?id=ba62/PLOSOne_formatting_sample_title_authors_affiliations.pdf>

**RESPONSE**: We have followed those style requirements

2. There are several cases where P-values are reported as being equal to/ less than 0, which is not possible. Please correct and clarify. For more details please see here: [https://journals.plos.org/plosone/s/submission-guidelines.#loc-statistical-reporting](https://journals.plos.org/plosone/s/submission-guidelines.#loc-statistical-reporting).

**RESPONSE**: We are sorry for that inaccuracy. P-values are now correctly reported in Table 1

3. We note that you have stated that you will provide repository information for your data at acceptance. Should your manuscript be accepted for publication, we will hold it until you provide the relevant accession numbers or DOIs necessary to access your data. If you wish to make changes to your Data Availability statement, please describe these changes in your cover letter and we will update your Data Availability statement to reflect the information you provide.

**RESPONSE**: We will provide repository information for our data at acceptance.

4. Please include your tables as part of your main manuscript and remove the individual files. Please note that supplementary tables (should remain/ be uploaded) as separate "supporting information" files.

**RESPONSE**: Tables are included as part of our main manuscript.

Additional Editor Comments:

This is an interesting paper examining the relationship between rumination, functions of reminiscence and autobiographical memory in younger and older adults. To my eye, it currently seems to contain a number of errors. In my opinion, it is not currently suitable for publication in this journal. However, it may be suitable for publication with some careful revision.

Please see below for my specific comments on this article (see also suggestions from reviewer 1):

1. With regard to recruitment of participants: were there any exclusion/inclusion criteria?

**RESPONSE**: This information has been included in “participants” section: Those participants who reported receiving treatment for mental illness were discarded. In addition, the group of older participants completed the Mini_Mental Status Examination (Lobo et al., 1979) to discard cognitive impairment.

2. ANOVA is mentioned in the results, though I do not see mention of this in the statistical analysis section of the materials and methods.

**RESPONSE**: We are sorry for this mistake.T-Tests were carried out instead of ANOVAs

3. Table 1. p values should not be less than .000. I believe this should be less than .001

**RESPONSE**: Totally agree, sorry for this mistake. Corrections have been yellow highligthed in yellow.

4. Table 2. Clarify what the double asterisks mean in terms of significance

**RESPONSE**: Now it reads: * = p < .01

5. Figures 1 and 2 seem to have the same caption. Should one of them be for older adults rather than younger?

**RESPONSE**: Sorry again for this mistake. Figure 1 is for young participants while Figure 2 is for older adults

6.In the discussion, is the idea of better emotional adjustment related to the younger participants having lower depression scores, despite a higher level of depressive rumination? I would add a caveat in discussion about the Beck Depression Inventory including items (changes in sleep, sex drive, appetite) that may differ between age groups for multiple reasons other than depression.

**RESPONSE:** Thank you for this nice idea. We have included it in discussion as follows: “However, this last finding should be interpreted carefully as the reasons to score higher in BDI in older adults compared to young adults could be due to the presence in the scale of items related to sleep, sex drive or appetite. Thus, a higher BDI score in older adults than in young participants is not necessarily an indicator of depression.”

7. The discussion also claims the current paper “lacks a sample with depressed participants”, but looking at the table it seems that at least some participants might be at least mildly depressed. The authors may wish to look at effects of excluding participants above cut-off points for mild depression on their analysis, or at least alter the language in the discussion so that it could not be misinterpreted as suggesting that people with depression were excluded from participation.

**RESPONSE:** We discarded clinically diagnosed with depression participants. We have clarified it as follows: “…the lack of a sample with clinically diagnosed depressed participants…”

Reviewer #1:

This article reports age differences between young and older groups in the effects of rumination and reminiscence on mood, specifically depression and anxiety. The study is well conducted and executed, with appropriate methodologies to the hypotheses presented. The results support the conclusions, and the findings will be of relevance to those using reminiscence therapy with older healthy or memory-impaired groups.

1. Overall I have no major changes or corrections to recommend; the writing is clear, but contains a very small number of phrasing issues (e.g. "equation structural model" instead of "structural equation model"); suggest a proofread by a native English speaker to address these minor problems.

**RESPONSE**: Now it reads: “structural equation model”. One of the co-authors is a native English speaker.

2. I was a little surprised to see little mention of the research showing cognitive and/or psychological health benefits of reminiscence therapy in various contexts. While this is not essential, it might be something for the authors to consider.

**RESPONSE:** Information on benefits of reminiscence therapy has been added in introduction section.

3.A recent paper by Allen et al. (2018) in Ageing Research Reviews may be of interest, as the authors outline a mechanism whereby emotional effects may contribute to cognitive gains. However, this should be considered an optional inclusion.

**RESPONSE**: The paper by Allen et al., (2018) has been included in discussion.

**RESPONSE TO REVIEWERS AND EDITOR IN THIS SECOND ROUND**

Many thanks to the authors for their response to the reviewers’ comments. The authors have addressed some of the reviewer comments adequately, but in the cases described below I’m not sure they have fully addressed the queries (see counter-responses). Overall, I feel that even after revision the quality of the manuscript is not quite tight enough for PLOS ONE. I’m sorry it is not better news. I do hope the authors have found the feedback useful, and I wish them best of luck in finding a publication outlet for their research on this interesting topic.

Additional Editor comments:

4. Table 2. Clarify what the double asterisks mean in terms of significance
**RESPONSE**: Now it reads: * = p < .01
**COUNTER-RESPONSE:** The body of the table contains double asterisks only, but only a single asterisk is given to signify P < .01 below the table?

**AUTHORS COUNTER-RESPONSE:** Now it reads: ** = p <.01

6. In the discussion, is the idea of better emotional adjustment related to the younger participants having lower depression scores, despite a higher level of depressive rumination? I would add a caveat in discussion about the Beck Depression Inventory including items (changes in sleep, sex drive, appetite) that may differ between age groups for multiple reasons other than depression.

**RESPONSE**: Thank you for this nice idea. We have included it in discussion as follows: “However, this last finding should be interpreted carefully as the reasons to score higher in BDI in older adults compared to young adults could be due to the presence in the scale of items related to sleep, sex drive or appetite. Thus, a higher BDI score in older adults than in young participants is not necessarily an indicator of depression.”

**COUNTER-RESPONSE:** The authors haven’t addressed the first part of this comment, i.e. clarifying whether emotional adjustment is operationalised as lower depression scores, despite higher level of depressive rumination.

**AUTHORS COUNTER-RESPONSE:** Now it reads: “Emotional adjustment was operationalized as lower depression scores despite higher levels of depressive rumination compared to older participants.”

7. The discussion also claims the current paper “lacks a sample with depressed participants”, but looking at the table it seems that at least some participants might be at least mildly depressed. The authors may wish to look at effects of excluding participants above cut-off points for mild depression on their analysis, or at least alter the language in the discussion so that it could not be misinterpreted as suggesting that people with depression were excluded from participation.

**RESPONSE:** We discarded clinically diagnosed with depression participants. We have clarified it as follows: “…the lack of a sample with clinically diagnosed depressed participants…”
**COUNTER-RESPONSE:** I do think the authors could have done more with regard to this issue (e.g. screening for depression, an analysis excluding participants above cut-off points), particularly as depression often goes undiagnosed.

**AUTHORS COUNTER-RESPONSE:** BDI scores were used as continuos dependent variable in the path models and not with a diagnostic purpose.

Reviewer #1
… suggest a proofread by a native English speaker to address these minor problems.
**RESPONSE:** Now it reads: “structural equation model”. One of the co-authors is a native English speaker.
**COUNTER-RESPONSE:** The English is still occasionally quite off/awkward to read, for example:
“From a developmental perspective, young adults need more making meaning and learning from experiences to develop emotional intelligence and consolidate new recent acquired relationships. On the contrary, previous experience in life have helped older adults to become more efficient in terms of emotional regulatory strategies and social rewarding needs.”
“the lack of a sample with clinically diagnosed depressed participants make also more difficult to extrapolate the current result to clinical samples.”

**AUTHORS COUNTER-RESPONSE:** The text has been proofread by a native English speaker.
