# Supplementary material for: Ruminative and mood associations for age differences in social and directive reasons to think and talk about life experiences
Source: PLoS One. 2020 Jul 16;15(7):e0235378. doi: 10.1371/journal.pone.0235378 (PMC7365414; doi:10.1371/journal.pone.0235378)
Supplement: Peer review history

# Author comment: Author response to previous submission

**DOI:** 10.1371/journal.pone.0235378.r001

21 May 2020

Attachment Submitted filename: Response to Reviewers 1.docx Click here for additional data file.

# Editor report: Decision Letter 0

**DOI:** 10.1371/journal.pone.0235378.r002

**Authors:** Andrew Allen

16 Jun 2020

Ruminative and mood associations for age differences in social and directive reasons to think and talk about life experiences

PONE-D-20-15235

Dear Dr. Jorge,

We are pleased to inform you that your manuscript has been judged scientifically suitable for publication and will be formally accepted for publication once it complies with all outstanding technical requirements.

Within one week, you will receive an e-mail containing information on the amendments required prior to publication. When all required modifications have been addressed, you will receive a formal acceptance letter and your manuscript will proceed to our production department and be scheduled for publication.

Shortly after the formal acceptance letter is sent, an invoice for payment will follow. To ensure an efficient production and billing process, please log into Editorial Manager at https://www.editorialmanager.com/pone/, click the "Update My Information" link at the top of the page, and update your user information. If you have any billing related questions, please contact our Author Billing department directly at authorbilling@plos.org.

If your institution or institutions have a press office, please notify them about your upcoming paper to enable them to help maximize its impact. If they will be preparing press materials for this manuscript, you must inform our press team as soon as possible and no later than 48 hours after receiving the formal acceptance. Your manuscript will remain under strict press embargo until 2 pm Eastern Time on the date of publication. For more information, please contact onepress@plos.org.

With kind regards,

Andrew Allen

Academic Editor

PLOS ONE
